# Long-Lasting Stable Expression of Human LL-37 Antimicrobial Peptide in Transgenic Barley Plants

**DOI:** 10.3390/antibiotics10080898

**Published:** 2021-07-23

**Authors:** Malihe Mirzaee, Edita Holásková, Alžbeta Mičúchová, David J. Kopečný, Zhila Osmani, Ivo Frébort

**Affiliations:** 1 Centre of Region Haná for Biotechnological and Agricultural Research, Czech Advanced Technology and Research Institute (CATRIN), Palacký University, 783 71 Olomouc, Czech Republic; malihe.mirzaee@upol.cz (M.M.); edita.holaskova@upol.cz (E.H.); alzbeta.micuchova@upol.cz (A.M.); zhila.osmani@upol.cz (Z.O.); 2 Department of Experimental Biology, Faculty of Science, Palacký University, 783 71 Olomouc, Czech Republic; d.kopecny@upol.cz

**Keywords:** barley, antimicrobial peptides, recombinant protein, field-grown progenies, LL-37

## Abstract

Antimicrobial peptides play a crucial role in the innate immune system of multicellular organisms. LL-37 is the only known member of the human cathelicidin family. As well as possessing antibacterial properties, it is actively involved in various physiological responses in eukaryotic cells. Accordingly, there is considerable interest in large-scale, low-cost, and microbial endotoxin-free production of LL-37 recombinant peptides for pharmaceutical applications. As a heterologous expression biofactory, we have previously obtained homologous barley (*Hordeum vulgare* L.) as an attractive vehicle for producing recombinant human LL-37 in the grain storage compartment, endosperm. The long-term stability of expression and inheritance of transgenes is necessary for the successful commercialization of recombinant proteins. Here, we report the stable inheritance and expression of the LL-37 gene in barley after six generations, including two consecutive seasons of experimental field cultivation. The transgenic plants showed normal growth and remained fertile. Based on the bacteria viability test, the produced peptide LL-37 retained high antibacterial activity.

## 1. Introduction

Nowadays, microbial infections are among the biggest threats to global public health. Moreover, the widespread administration of conventional antibiotics in recent decades has resulted in a notable increase in drug-resistant microbes. Antimicrobial peptides (AMPs), as novel potential alternatives to antibiotics, are essential components of the innate immune system, exhibiting a wide range of antimicrobial activities. AMPs have been found in virtually all living creatures; in plants, they act by defending against bacterial and fungal invasion, whereas in vertebrates and invertebrates they also exhibit activity against viral and parasitic infections. Although AMPs differ in their length, composition, and secondary structure, they generally contain fewer than 50 amino acids, are positively charged, and can adopt amphipathic structures. These peptides, without high-specificity, target protein-binding sites, reduce the likelihood of induced microbial resistance. Therefore, it seems that AMPs can be used as new potential therapeutic agents to overcome microbial drug resistance [1,2,3,4,5,6,7]. They are also especially suited to the transdermal route of administration.

Among the hundreds of synthesized AMPs present in humans, LL-37 is the single representative of the cathelicidin family. A cationic AMP, LL-37, has an alpha-helical structure and amphipathic properties, which lead to its interaction with anionic lipids of the bacterial membrane or other negatively charged components. This 37-residue peptide has gained increased attention because of its multifactorial mode of action [8,9]. Originally identified for its broad-spectrum antimicrobial activity against bacteria, fungi, and viruses, LL-37 also has been associated with important physiological functions in chemotaxis, stimulation of wound closure, regulation of the inflammatory response, apoptosis, angiogenesis, and cancer tumorigenesis [10,11,12]. A novel therapeutic based on an LL-37 named ropocamptide, which is under development by Promore Pharma AB for the healing of venous leg ulcers and possibly also diabetic foot ulcers, has recently passed a phase IIb clinical trial in Sweden and Poland [13,14]. Thus, the production of biologically active LL-37, with high yield and low cost, is essential for biomedical applications.

Foreign proteins can be synthesized in a broad spectrum of host organisms, including bacterial, yeast, insect, plant, and mammalian cells [15,16,17]. Several approaches have been reported for the production of recombinant LL-37 in bacteria, i.e., *Escherichia coli* (*E. coli*) [18,19,20,21,22]. However, endotoxins are common contaminants originating in Gram-negative bacteria, and since it is difficult to completely remove them from biological material, they have long been considered the most detrimental threats to commercially bacterial-produced bioproducts. As heterologous expression biofactories, plants offer specific advantages over other eukaryotic or even prokaryotic systems in terms of large-scale and economical production of therapeutic proteins. Moreover, as intrinsically safe organisms, plants contain no endotoxins or replicating human pathogens. Plants also generally allow recombinant proteins from other organisms to be synthesized while retaining their proper folding and activity [23,24,25,26,27].

In planta-produced AMPs have not only exhibited antimicrobial activities [28,29,30,31,32], but have also been noted for their ability to enhance the disease resistance of transgenic plants [33,34,35,36]. Recombinant production of LL-37 and its variants in plant systems has been reported with the aim to confer resistance to several bacterial and fungal pathogens in Chinese cabbage [37], tomato [38], and rice [39]. Our group has recently established a viable approach using molecular farming for the production of recombinant human LL-37 (rhLL-37) in barley grains [40].

Currently, tobacco (*Nicotiana tabacum*) and its close relative benth (*Nicotiana benthamiana*), as non-food/feed leafy crops, are the most commonly used plant-based platforms for heterologous protein production. However, their usage is restricted by the high content of phenolic compounds and undesired proteolytic enzymes, which must be completely removed in downstream processing steps [41,42,43,44]. By contrast, a seed-based bioreactor system provides an appropriate biochemical and physiological environment for the long-term storage of pharmaceutical proteins. In the present study, we investigated the stable inheritance, consistent expression, and bioactivity of recombinant rhLL-37 fused with maltose-binding protein (MPB) in the sixth generation of transgenic barley; the study included two consecutive seasons of growth in an agricultural field.

## 2. Results

### 2.1. Confirmation of MBP::rhLL-37 Gene Insertion in Transgenic Progeny Barley Plants

**Figure 1:**
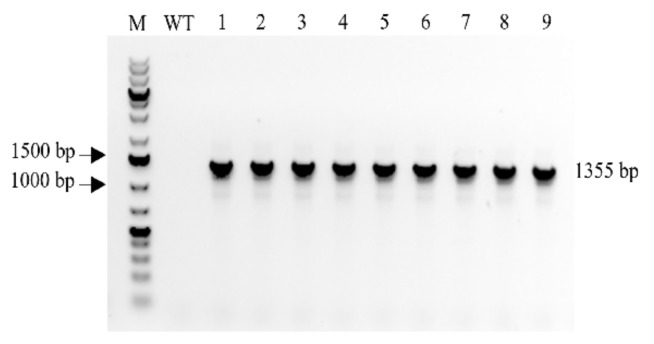
PCR amplification of a 1355 bp fragment of *MBP::rhLL-37* gene from barley genomic DNA. M, 1 kb plus DNA ladder; WT, Wild type (non-transformed barley plant); lane 1, T5 and lanes 2–9, T6 progenies of a homozygous MBP::rhLL-37 transgenic barley line.

After obtaining T0 transgenic barley plants with a stably integrated *MBP::rhLL-37* fusion gene [40], the homozygous line with a single copy of T-DNA insert was subsequently used for producing progeny plants in the greenhouse and finally in the field (Supplementary Figure S1). The presence of the transgene in the field-grown transgenic generations of barley plants was confirmed by a genomic PCR screening, which showed the expected 1355 bp bands. On the other hand, there was no such band detected in the wild-type control plant samples (Figure 1).

### 2.2. Detection of the MBP::rhLL-37 Gene Transcripts in Progeny Barley Grains

**Figure 2:**
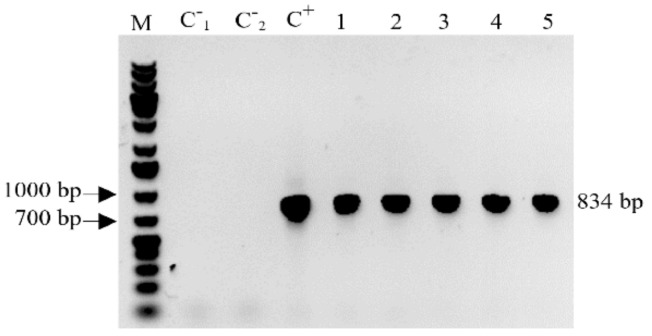
RT-PCR amplification of an 834 bp fragment from *MBP::rhLL-37* gene transcript. M, 1 kb plus DNA ladder; C^−^_1_, negative control (RNA template); C^−^_2_, negative control (non-transformed plant); C^+^, positive control (gDNA of MBP::rhLL-37 transgenic barley plant); lane 1, T5 and lanes 2–5, T6 progenies of MBP::rhLL-37 transgenic barley plants.

A reverse transcription-PCR (RT-PCR) analysis was performed to determine transcription of the *MBP::rhLL37* transgene in the samples from randomly selected field-grown progenies. The mRNA extracts from transgenic barley plants all showed an 834 bp band; however, no signal was observed in the samples from the non-transformed plants used as a negative control (Figure 2).

### 2.3. Detection and Quantification of the MBP::rhLL-37 Fusion Protein in Field-Grown Barley Plants

**Figure 3:**
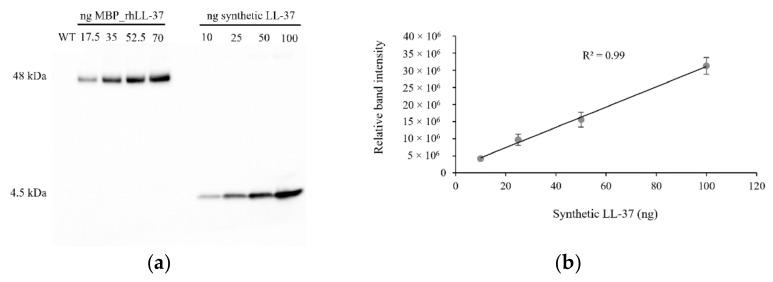
Quantification of the MBP_rhLL-37 fusion protein. (**a**) Immunoblot analysis of crude extracts of lyophilized samples from transgenic T6 field-grown barley plants expressing endosperm-targeted fusion protein MBP_rhLL-37 in the late milk endosperm seeds with LL-37 specific antibody; WT, wild type (non-transformed plant). (**b**) The calibration curve of synthetic LL-37 peptide was used for semiquantitative estimation.

The recombinant MBP_rhLL-37 fusion protein in transgenic barley plant extracts was detected by immunoblot with the LL-37 monoclonal IgG1 antibody. A 48 kDa band of the expected MBP_rhLL-37 protein was observed in the samples from transgenic progenies, whereas the non-transformed plant samples did not show any signal, indicating that the LL-37 IgG1 antibody did not cross-react with common barley grain proteins. The expression level of the fusion protein was quantified using the semiquantitative method. Based on a dilution series of synthetic LL-37 as a positive control, the accumulation was up to 250 ± 5.36 mg of rhLL-37 per kilogram of lyophilized spike tissue in the field-grown T6 generation MBP::rhLL-37 plants (Figure 3); this was about 3.5 times more than non-lyophilized samples (the difference roughly accounts for the loss of water during lyophilization), and at the same level as in the T5 generation (Supplementary Figure S2). It needs to be mentioned that since in a large-scale preparation the separation of the endosperm from each grain is technically impossible, protein extraction must be performed from the whole spike, where the endosperm tissue accounts for around 35% of the spike weight in the late milky stage (BBCH 77).

### 2.4. Analysis of the Antibacterial Activity of rhLL-37 from Barley

**Figure 4:**
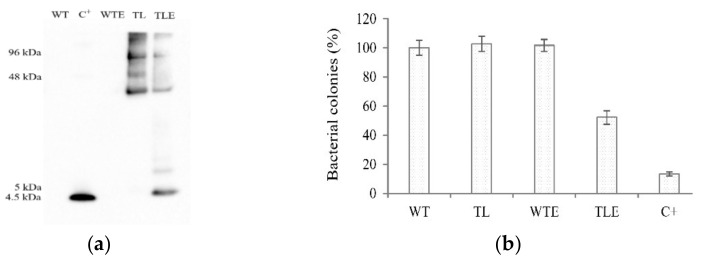
Analysis of the antibacterial activity of rhLL-37. (**a**) Immunoblot analysis of enterokinase-released rhLL-37 peptide purified by amylose resin affinity chromatography from late milk endosperm grains (BBCH 77) of MBP::rhLL-37 transgenic T6 generation barley plants. The sizes of individual bands corresponded to the theoretical size of the MBP_rhLL-37 fusion peptide (48 kDa), dimer aggregate (96 kDa), and enterokinase released rhLL-37 (5 kDa), or synthetic LL-37 peptide (4.5 kDa). Proteins from wild type (WT, non-transformed plant) and transgenic line (TL) subjected to enterokinase cleavage are indicated as WTE and TLE, respectively. A total amount of 100 ng synthetic LL-37 (C^+^) and 200 ng of recombinant peptides was loaded on the gel. (**b**) Antibacterial activity analysis of the purified samples prepared from late milk endosperm grains (BBCH 77) of T6 generation of transgenic barley. The biological activity was examined with *E. coli* TOP10, which was mixed with 400 ng of either synthetic LL-37 or rhLL-37. The number of bacterial colonies on agar plates formed in the presence of purified protein extracts from wild type barley grains was considered as 100%. The data represent the means of three independent experiments, each in three technical replicates.

MBP_rhLL-37 fusion has been designed in such a way that enterokinase cleavage leaves no additional residues at the N-terminus of rhLL-37 [40]. However, compared to the native human LL-37 peptide, rhLL-37 contains an additional C-terminal endoplasmic reticulum retention signal composed of the four amino acids, KDEL. To elucidate the antibacterial activity of the rhLL-37 peptide extracted from transgenic field-grown barley progenies, samples containing the amylose-resin-purified full-length MBP_rhLL-37 fusion peptide, as well as their enterokinase-treated aliquots, were examined. Under the experimental conditions, the efficiency of the cleavage by enterokinase was around 50% (Figure 4a). As shown in Figure 4b, the inhibition of bacterial growth was observed after the addition of the synthetic LL-37 or the purified rhLL-37 peptide, whereas the MBP_rhLL-37, which was not treated with enterokinase or extracts from wild-type plants, did not exert any inhibitory effect on the growth of *E. coli*. These results confirmed the antibacterial activity of the in planta-produced rhLL-37 peptide in advanced generations of transgenic barley. These findings were similar to the results obtained from the early progenies [40], in which the antimicrobial activity of rhLL-37 peptide was retained even after adding the endoplasmic reticulum (ER) retention signal, KDEL, at the C-terminus.

As shown in Figure 4a (lanes TL and TLE), bands with higher molecular mass than was expected for MBP_rhLL-37 were detected with the LL-37 antibody. MBP alone is known to be a monomeric molecule, although some MBP fusions with other proteins have been shown to form dimers, tetramers, or larger forms due to their fused protein features [45,46,47]. As reported previously, LL-37 peptides can form oligomers by hydrophobic interactions [48,49,50]. Therefore, the oligomerization of MBP_rhLL-37 at the higher concentrations used for the cleavage experiment (Figure 4a) is probably brought about by the rhLL-37-peptide part of the MBP_rhLL-37 fusion protein.

## 3. Discussion

One of the most important cereals worldwide, barley (*Hordeum vulgare* L.) can be cultivated across a broader variety of climates than any other cereal. The use of barley grains as a desirable bioreactor has been developed for the production of human therapeutic proteins because the endosperm of barley grain is a natural storage site for the accumulation of proteins. Within the developing endosperm, ER chaperones and disulfide isomerases facilitate protein folding and maturation, which can be applied for the production of high-quality recombinant transgenes [27,51,52,53]. The recombinant protein will thereby be placed in a suitable environment with high folding and storage capacity and low protease activity. Moreover, downstream processing of the heterologous products is largely assisted by the fact that barley grain provides an endotoxin-free environment with relatively low secondary metabolite content; barley grain also has a relatively simple protein profile. On the other hand, the infrastructures for large-scale grain production, harvest, transport and post-harvest storage are well-established. Additionally, cultivated diploid barley is strictly self-pollinated with no outcrossing reducing the possibility of gene drift. Accordingly, barley received the GRAS (generally regarded as safe) status from the FDA [54,55,56,57].

Our group has shown that the human cathelicidin can be efficiently produced in barley grains. However, in contrast to our previous report [40], here we have estimated the accumulation of MBP fused to rhLL-37 in barley grains in crude protein extracts before purification, in serial dilutions of total soluble proteins (TSPs), and in instances without heating prior to semiquantitative immunoblot analysis, which all led to a largely improved assessment of the MBP_rhLL-37 expression level. As the MBP domain could stabilize and protect the rhLL-37 peptide against degradation together with its affinity-purification amenability, we have chosen the MBP_rhLL-37 fusion protein for multigenerational assessments. For the time being, barley grains have also been applied as a superior host for the production of human therapeutics such as human α1-antitrypsin, serum albumin, antithrombin III and lysozyme [58], human lactoferrin [59,60], full-length collagen type I α1 (rCIa1) [54,61], human growth factor FLT3 ligand [62], and HIV-neutralizing 2G12 antibody [63,64]. Moreover, ORF Genetics Iceland used the great potential of barley grains in producing cytokines and human-like growth factors to commercialize over 40 products in the dermatology, cosmetic and personal care areas (https://www.orfgenetics.com/, accessed on 21 June 2021)), demonstrating that barley is a realistic platform for the production of endotoxin-free proteins.

The commercialization of a transgenic crop highly depends upon the stable and predictable expression of the introduced gene, and the absence of silencing during long-term generation advances. In this way, we confirmed the stable *MBP::rhLL-37* gene inheritance and its consistent expression without silencing in homozygous barley progenies that were advanced to T5 and T6 field cycles. Only transgenic barley plants harboring a single copy number of the *MBP::rhLL-37* gene, without changes in phenotype, were selected for developing the subsequent generations. Although multiple copies of transgenes can be integrated at once into a plant genome via genetic transformation, stacking several copies of foreign genes in that way is unreliable since they may not be equally stably distributed through generations and/or their expression might not be equal. Therefore, transgenic lines harboring single transgene or low copy numbers are less prone to exhibit transgene silencing and thus are more desirable for molecular farming [65,66]. The other possible reasons for the silencing of a foreign gene could be promoter interference and methylation of the first untranslated exon and 5′ end of the intron in the constitutive ubiquitin promoter complex [67,68]. In barley, it has been shown that the long-term stability of transgene expression could be derived by using endosperm-specific promoters, whereas comparable expression driven by the frequently used consecutive maize-ubiquitin promoter often leads to expression loss in earlier generations [69,70,71,72,73,74]. Moreover, in many cases, the transcriptional nature of constitutive promoters often leads to poor growth of the host plants and affects yields, whereas seed-specific promoters restrict the accumulation of heterologous proteins in seed tissue. Therefore, in addition to the increased stability of the recombinant protein, the vegetative organs are preserved from the undesirable effects of accumulated foreign proteins [75,76,77,78]. In this context, our transgenic barley plants, which stably expressed rhLL-37 driven by the barley endosperm-specific B1 hordein gene promoter, illustrate the functional expression of transgenes in progeny up to the T6 generation.

Recently, Takaiwa et al. (2021) [79] reported that the accumulation of the human transforming growth factor-β1 in transgenic rice grains could be boosted to an extraordinarily high level of 452 μg/grain by using a 26 kDa α-globulin endosperm-specific promoter along with the simultaneous reduction in major endogenous seed storage proteins through RNAi. In barley, Panting et al., (2021) [57] demonstrated an improvement in the yield of the human epidermal growth factor through targeted mutation of the B-hordein storage protein, which strongly reduced the storage protein content and increased target recombinant peptide production. We believe that, as the next step, the custom peptide synthesis could be accelerated by replacing the abundant B-hordein seed storage protein with the peptide driven by the promoter of the suppressed gene.

In conclusion, barley grains, unlike bacteria, provide an endotoxin-free platform for the production of high-value therapeutic proteins. Our group has previously expressed the rhLL-37 [40], β-defensin 2, and pexiganan [80] in endosperms of the transgenic barley seeds. In the current study, we observed high stability and inheritance of the *rhLL-37* transgene expression driven by the barley B1 hordein promoter up to the T6 generation in the field trials. This is vital for the successful commercialization of heterologous proteins produced in a plant-based bioreactor. The advanced generations of transgenic barley showed normal growth and the produced rhLL-37 retained high antibacterial activity.

## 4. Materials and Methods

### 4.1. Plant Material and Growth Conditions

The transgenic MBP::rhLL-37 Golden Promise barley plants obtained by *Agrobacterium*-mediated transformation as previously described [40] were used for field trials. The T5 and T6 generation plants selected from a single homozygous T1 line were cultivated under the permission ref. no. MZP/2018/750/2786 from the Ministry of Environment, Czech Republic, in a GM plant experimental facility (operated by the company Úsovsko, Klopina, Czech Republic), in two consecutive seasons, 2019 and 2020. The spikes were harvested by hand at the late milk development stage (BBCH 77), corresponding to the highest amount of MBP_rhLL-37 in the barley grains. The spikes were then lyophilized, ground, and stored at −20 °C for subsequent assessments.

### 4.2. Polymerase Chain Reaction (PCR) Analysis of the Transgene Insertion

The total genomic DNA was extracted from the leaves of wild-type and transgenic barley plants using the phenol–chloroform method [81]. PCR was performed to confirm transgene presence, using genomic DNA (gDNA) with primers specific to hpt gene (5′-GAATTCAGCGAGAGCCTGAC-3′ and 5′-ACATTGTTGGAGCCGAAATC-3′), or *MBP::rhLL-37* gene fusion (5′-GCCGTGGTGTACTACCTCCTC -3′ and 5′-TCACAGCTCATCCTT GGACTC -3′). In each PCR reaction, after primary denaturation for 5 min at 98 °C, samples were subjected to 35 cycles of 30 s at 98 °C, 30 s at 54 °C and 30/90 s (for hpt, and *MBP::rhLL-37* elongation, respectively) at 72 °C followed by a final elongation for 5 min at 72 °C. The PCR products were analyzed by electrophoresis in a 1% (*w*/*v*) agarose gel.

### 4.3. Reverse Transcription-PCR of the Transgene Specific mRNA

Total RNA was isolated from the grains of transformed and untransformed barley grains in the late milk endosperm stage (BBCH 77) using a Quick-RNA Plant MiniPrep kit (Zymo Research). After removing genomic DNA by DNAseI (Thermo Fisher Scientific), complementary DNA (cDNA) was obtained from 2 μg of the total RNA samples by a First Strand cDNA Synthesis Kit (Thermo Fisher Scientific). RT-PCR was carried out on cDNA by specific primers (5′-AGGCCCTCTCCCTGATCTACA -3′ and 5′-GGACGATCCTCTTGAACTCCT -3′) located in the *MBP::rhLL-37* gene transcript sequence.

### 4.4. Extraction and Quantification of the Recombinant Protein

The barley grain powder (100 mg) was resuspended in 800 µL of protein extraction buffer (0.1 M Tris-HCl, pH 8.0, containing 0.3 M NaCl, 1 mM EDTA, 1 mM DTT, and 1 mM PMSF). The extraction was performed on an inverting rotator at 4 °C for 1 h. The samples were then centrifuged at 14,000× *g* for 20 min at 4 °C to eliminate the insoluble fraction. The concentration of TSPs was measured using the Bradford reagent (Bio-Rad), with bovine serum albumin (BSA) as standard. For the immunoblot analysis, TSP samples were separated under reducing conditions on 4–12% gradient Bis-Tris Plus precast polyacrylamide gels (Thermo Fisher Scientific). The electrophoresed proteins were subsequently transferred to a polyvinylidene difluoride (PVDF) membrane with a wet-transfer apparatus (Bio-Rad) for 16 h at a constant voltage of 20 V. The PVDF membrane was then soaked in iBind™ solution (Thermo Fisher Scientific) for 5 min at room temperature, after which Western blotting was performed using the iBind Western System (Thermo Fisher Scientific). The MBP_rhLL-37 protein was detected by the LL-37 mouse monoclonal IgG1 (sc-166770; Santa Cruz Biotechnology, Santa Cruz, CA, USA) at 1:400 dilution for 90 min. Immunodetection was performed for 90 min with a 1:1000 diluted m-IgGk BP-HRP anti-mouse secondary antibody (sc-516102; Santa Cruz Biotechnology, Santa Cruz, CA, USA) and enhanced chemiluminescent substrate (ECL kit; Bio-Rad). To estimate the accumulation level of rhLL-37 in barley grains, 10, 25, 50 and 100 ng of synthetic LL-37 (Sigma-Aldrich, Saint Louis, MO, USA) were used as standards for the semiquantitative analysis. The signal intensity was calculated with Image Lab Software (Bio-Rad) using three different biological replicates.

### 4.5. Antibacterial Activity Assay

The MBP_rhLL-37 fusion protein was purified on an amylose resin affinity chromatography column (New England Biolabs), and then cleaved by enterokinase (New England Biolabs) according to the manufacturer’s instructions. The released barley-derived rhLL-37 peptide was used for comparing the antibacterial activity with the synthetic LL-37; the sample from the non-transformed barley plant was used as a control using a modified version of the previously used protocol [40]. Briefly, a single colony of *E. coli* TOP10 was inoculated into 5 mL of LB liquid medium in a shaking incubator at 180 rpm and cultivated at 37 °C overnight. The following day, the culture was diluted in fresh LB medium, incubated at 37 °C, 180 rpm to the log phase (OD600 ~ 0.5), and then harvested by centrifugation for 5 min at 6000× *g*. Afterward, a standard cell suspension was prepared by resuspending the cell pellet in 5 mM ammonium bicarbonate (pH 7.4) resulting in a suspension containing 2 × 10^8^ colony-forming units (CFU) per mL. Next, purified rhLL-37 peptides were buffer-exchanged into 5 mM of ammonium bicarbonate (pH 7.4) and concentrated using 3 kDa cut-off Amicon centrifugal filters (Merck Millipore, Burlington, MA, USA) to a final concentration of 40 µg of protein/mL. Then, aliquots of 1 µL of the *E. coli* suspension were mixed with 10 µL of rhLL-37 peptide or synthetic LL-37, and the final volume was adjusted to 20 µL by adding 5 mM of ammonium bicarbonate (pH 7.4). Following incubation at room temperature for 1 h with continuous orbital shaking at 1000 rpm, the mixtures were 10^3^-fold diluted in 5 mM of ammonium bicarbonate (pH 7.4), and finally, their 100 µL aliquots were spread over LB agar plates and incubated for 24 h at 37 °C. The antimicrobial activity was determined as a percentage of bacterial colonies grown on the agar plates for rhLL-37 in comparison with controls. The assay was carried out in three independent experiments.

## 5. Conclusions

In conclusion, barley grains, unlike bacteria, provide an endotoxin-free platform for the production of high-value therapeutic proteins. Our group has previously expressed the rhLL-37 [40], β-defensin 2, and pexiganan [81] in endosperms of the transgenic barley seeds. In the current study, we observed high stability and inheritance of the *rhLL-37* transgene expression driven by the barley B1 hordein promoter up to the T6 generation in the field trials. This is vital for the successful commercialization of heterologous proteins produced in a plant-based bioreactor. The advanced generations of transgenic barley showed normal growth and the produced rhLL-37 retained high antibacterial activity.

## 6. Patents

Holásková, E., Galuszka, P., Frébort, I., Milek, J. (2019) A method of preparing barley plants that produce antimicrobial peptide. WO2019052588.

## Data Availability

Data are contained within the article or supplementary material.

## Supplementary Materials

The following are available online at https://www.mdpi.com/article/10.3390/antibiotics10080898/s1, Figure S1. Propagation of T6 generation of transgenic MBP::rhLL-37 Golden Promise barley plants in 200 m^2^ field trial in 2020. The average yield of the late milk endosperm seeds (BBCH 77) was 0.54 kg fresh weight/m^2^, corresponding to 90 g lyophilized material/m^2^. Figure S2. Immunoblot analysis of nonlyophilized samples from transgenic T5 and T6 field-grown barley plants expressing endospermtargeted MBP_rhLL-37 protein in the late milk endosperm seeds (BBCH 77). Expression levels of MBP_rhLL-37 in the barley grains were estimated by a semiquantitative analysis using the calibration curve with synthetized LL-37 peptide (Figure 3b). WT, wild type (non-transformed plant).
