# Supplementary material for: Long-Lasting Stable Expression of Human LL-37 Antimicrobial Peptide in Transgenic Barley Plants
Source: Antibiotics (Basel). 2021 Jul 23;10(8):898. doi: 10.3390/antibiotics10080898 (PMC8388648; doi:10.3390/antibiotics10080898)
Supplement: Supplementary file 1 [file antibiotics-10-00898-s001.zip › antibiotics-1289691-supplementary.pdf]

antibiotics

Article

Long-Lasting Stable Expression of Human LL-37 Antimicrobial Peptide in Transgenic Barley Plants

Malihe Mirzaee [1], Edita Holásková [1], Alžbeta Mičúchová [1], David J. Kopečný [2], Zhila Osmani [1] and Ivo Frébort [1,*]

[1] Centre of Region Haná for Biotechnological and Agricultural Research, Czech Advanced Technology and Research Institute (CATRIN), Palacký University, 783 71 Olomouc, Czech Republic; malihe.mirzaee@upol.cz (M.M.); edita.holaskova@upol.cz (E.H.); alzbeta.micuchova@upol.cz (A.M.); zhila.osmani@upol.cz (Z.O.)
[2] Department of Experimental Biology, Faculty of Science, Palacký University, 783 71 Olomouc, Czech Republic; d.kopecny@upol.cz
* Correspondence: ivo.frebort@upol.cz; Tel.: +420-585-634-922

Supplementary Materials

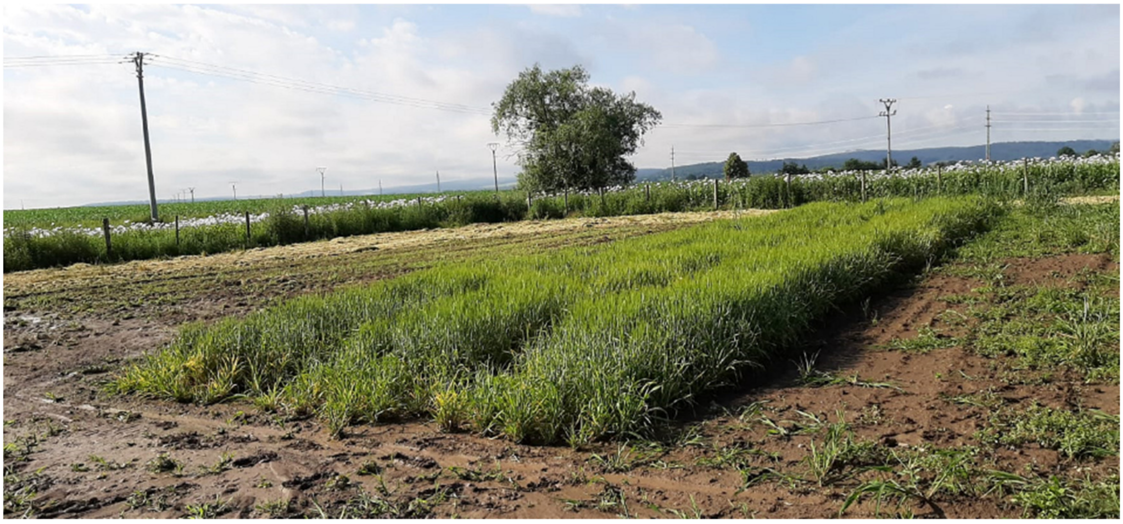

Figure S1. Propagation of T6 generation of transgenic MBP::rhLL-37 Golden Promise barley plants in 200 m² field trial in 2020. The average yield of the late milk endosperm seeds (BBCH 77) was 0.54 kg fresh weight/m², corresponding to 90 g lyophilized material/m².

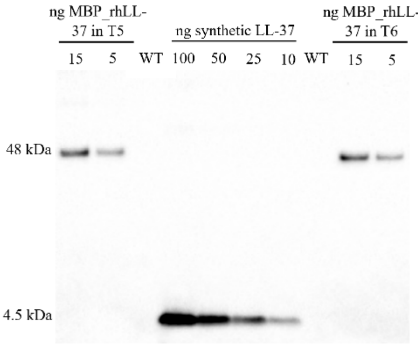

Figure S2. Immunoblot analysis of non-lyophilized samples from transgenic T5 and T6 field-grown barley plants expressing endosperm-targeted MBP_rhLL-37 protein in the late milk endosperm seeds (BBCH 77). Expression levels of MBP_rhLL-37 in the barley grains were estimated by a semi-quantitative analysis using the calibration curve with synthetized LL-37 peptide (Figure 3b). WT, wild type (non-transformed plant).
